# A literature review: mechanisms of antitumor pharmacological action of leonurine alkaloid

**DOI:** 10.3389/fphar.2023.1272546

**Published:** 2023-09-25

**Authors:** Qiang Cao, Qi Wang, Xinyan Wu, Qi Zhang, Jinghan Huang, Yuquan Chen, Yanwei You, Yi Qiang, Xufeng Huang, Ronggao Qin, Guangzhu Cao

**Affiliations:** ^1^ Department of Earth Sciences, Kunming University of Science and Technology, Kunming, China; ^2^ School of Medicine, Macau University of Science and Technology, Taipa, China; ^3^ Department of Gastroenterology, Affiliated Hospital of Jiangsu University, Jiangsu University, Zhenjiang, China; ^4^ College of Veterinary Medicine, Sichuan Agricultural University, Chengdu, China; ^5^ Undergraduate Department, Taishan University, Taian, China; ^6^ Undergraduate Department, Sichuan Conservatory of Music, Chengdu, China; ^7^ Institute of Medical Information/Library, Chinese Academy of Medical Sciences, Beijing, China; ^8^ Division of Sports Science and Physical Education, Tsinghua University, Beijing, China; ^9^ Faculty of Dentistry, University of Debrecen, Debrecen, Hungary

**Keywords:** leonurine, anti-tumor mechanisms, signal transduction pathways, apoptosis and autophagy, tumor cell proliferation and migration

## Abstract

Leonurine refers to the desiccated aerial portion of a plant in the Labiatae family. The primary bioactive constituent of Leonurine is an alkaloid, Leonurine alkaloid (Leo), renowned for its substantial therapeutic efficacy in the treatment of gynecological disorders, in addition to its broad-spectrum antineoplastic capabilities. Over recent years, the pharmacodynamic mechanisms of Leo have garnered escalating scholarly interest. Leo exhibits its anticancer potential by means of an array of mechanisms, encompassing the inhibition of neoplastic cell proliferation, induction of both apoptosis and autophagy, and the containment of oncogenic cell invasion and migration. The key signal transduction pathways implicated in these processes include the Tumor Necrosis Factor-Related Apoptosis-Inducing Ligand (TRAIL), the Phosphoinositide3-Kinase/Serine/Threonine Protein Kinase (PI3K/AKT), the Signal Transducer and Activator of Transcription 3 (STAT3), and the Mitogen-Activated Protein/Extracellular Signal-Regulated Kinase (MAP/ERK). This paper commences with an exploration of the principal oncogenic cellular behaviors influenced by Leo and the associated signal transduction pathways, thereby scrutinizing the mechanisms of Leo in the antineoplastic sequence of events. The intention is to offer theoretical reinforcement for the elucidation of more profound mechanisms underpinning Leo’s anticancer potential and correlating pharmaceutical development.

## 1 Introduction

At present, the global escalation of both incidence and mortality rates of cancer is swiftly becoming a substantial menace to human health. A multitude of natural products, particularly small natural molecules, often exhibit potent anticancer activities whilst maintaining low toxicological side-effects. These qualities render them paramount in the process of antitumor drug screening, thus prompting an upsurge of interest from the scientific community (Dehelean et al., 2021; Hashem et al., 2022; Liang et al., 2020; Majolo et al., 2019; Zhu et al., 2022). Leonurine, a biologically active alkaloid with diverse pharmacological properties, is derived from the desiccated aerial portion of plants belonging to the Labiatae family, commonly known as the mint family. These plants, including species like Leonurus cardiaca (Motherwort), serve as the primary source of leonurine alkaloid.

Leonurine alkaloid, the primary active biological alkaloid contained within Motherwort, has been acknowledged for its extensive array of biological functions and its exceptional antitumor properties (Zhu et al., 2018; Leorita et al., 2020; Liu et al., 2021; Liang et al., 2022). The cornerstone of Leo’s antitumor actions primarily hinge upon its anti-inflammatory and antioxidant activities (Lin et al., 2020; Chen et al., 2023; Tian et al., 2023). Additionally, Leonurine exhibits capacity to impede tumor cell cycles, induce apoptosis in tumor cells, and stimulate the immune system.

Emerging from Zhang et al.’s study (Zhang et al., 2019), it has been posited that Leonurine alkaloid moderates the progression of hepatocellular carcinoma in murine models via the AMPK/SREBP-1c signaling pathway. In addition to its role in moderating hepatocellular carcinoma, Leo’s impact on oxidative stress and cancer risk reduction is noteworthy. Leo’s antioxidative properties are underpinned by its ability to enhance cellular oxidative stress responses and bolster superoxide dismutase (SOD) activity. Oxidative stress is a critical factor in cancer development, as it contributes to DNA damage and genomic instability, potentially leading to tumorigenesis. Leo’s capacity to enhance SOD activity, which is essential for mitigating cellular oxidative damage, has significant implications for reducing the risk of cancer initiation and progression. By bolstering the cellular defense against oxidative stress, Leo showcases its potential not only in impeding cancer progression but also in preemptively reducing the chances of cancer development. Shen et al.’s study (Shen et al., 2023) posits that the Leonurine alkaloid can mitigate lung cancer through the NF-κB signaling pathway, decrease phosphorylation and nuclear translocation in myocardial cells’ P65, and subsequently attenuate the expression of inflammatory factors such as IL-6, TNF-α, and MCP-1. Deng et al.’s study (Deng et al., 2022) unveils that Leonurine alkaloid can ameliorate cellular oxidative stress, enhance SOD activity, thus diminishing the risk of cancer.

There are myriad studies suggesting that Leo can halt cancer progression or manifest anticancer effects (Chen et al., 2021; Qiang et al., 2022; Raza et al., 2022; Ajzashokouhi et al., 2023; Chi et al., 2023). This article undertakes a review of the research concerning the anticancer pharmacological mechanisms of Leo, thereby facilitating further insights for pharmacological research and novel drug development.

## 2 Main antitumor mechanisms of leo

**FIGURE 1:**
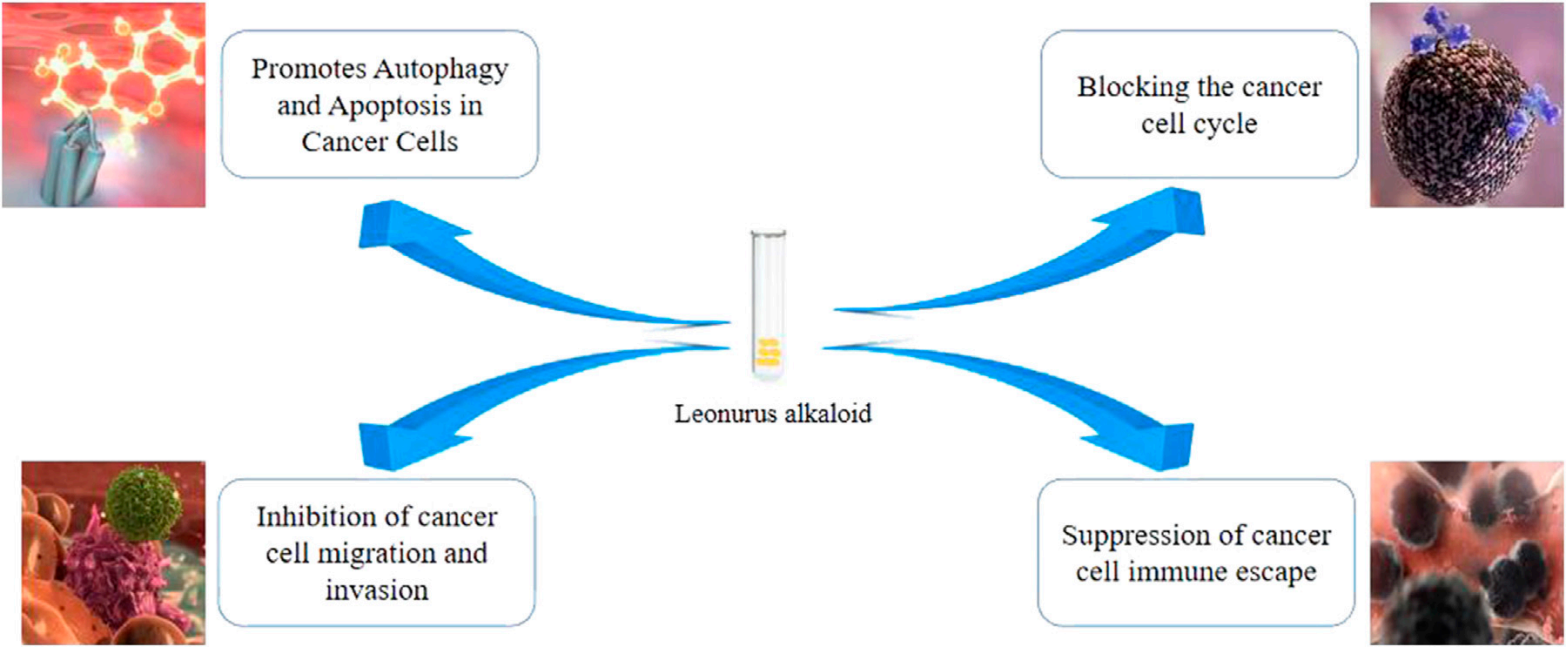
Mechanism of antitumour action of Leonurine alkaloid.

Leo displays antitumor effects both *in vivo* and *in vitro*, with physiological effects as shown in Figure 1. It exerts its antitumor actions by inducing tumor cell apoptosis and inhibiting tumor cell proliferation, invasion, and migration (Liu et al., 2018; Hu et al., 2019; Zhuang et al., 2021).

### 2.1 Leo promotes tumor cell cycle arrest

The complex orchestration of cellular cycle modulation is effectuated through a sophisticated and intricate network of interdependencies, which encompass an assortment of proteins, enzymes, cytokines, and cell cycle signaling pathways. These components are fundamentally pivotal to the execution of cellular proliferation, growth, and regeneration processes. The cell cycle itself is partitioned into four cardinal phases, namely the first interphase gap (G1), the DNA synthesis phase (S), the second interphase gap (G2), and the mitotic phase (M) (Zou and Lin, 2021; You et al., 2023a; Yuan et al., 2023). Deviations in the precision of this cellular cycle regulation demonstrate a close association with the onset, advancement, and distant metastasis of tumorous growths (Wang, 2021; Zou and Lin, 2021; You et al., 2023b). As a result, modulating cell cycle distribution and inducing cellular cycle arrest are perceived as potent methodologies for anticancer interventions (Bonelli et al., 2019; Guan and Guan, 2020; Cao et al., 2022a). Leo can exert its antiproliferative impact on malignant cells by mediating the regulation of cell cycle and cellular proliferation processes. Leo can induce a state of cellular cycle arrest in cancerous cells, either at the G0/G1 or G2/M phase, which effectively inhibits cellular expansion. It negatively regulates the expression of cyclins D1 and D2, along with their partner proteins, Cyclin-Dependent Kinases 2 (CDK2), CDK4, and CDK6. Simultaneously, it increases the expression of p21WAE1/CIP1 and p27, thereby inhibiting the transition from G0 to G1 phase and the preparatory activities leading to the S phase, primarily through the control of protein levels (Chen et al., 2015; Chen et al., 2019; Xu et al., 2020; Cao et al., 2022b). Yu et al.’s study (Yu et al., 2023) found that Leo can maintain the Cyclin D1-CDK4 complex at lower levels by reducing the expression of CDK4, thereby stalling the cell cycle. In studies using Leo-treated breast cancer cell lines, it was found that the expression levels of Cyclins A1, B1, and CDK1 decreased in the treated cells, while p53 and BAX were upregulated, resulting in a significant cell cycle arrest at the G2/M phase, which subsequently leads to cell apoptosis (Chen et al., 2013a; Sitarek et al., 2021; Cao et al., 2022c; Xi et al., 2023).

Li et al.’s study (Li et al., 2020) showed that Leo has anti-inflammatory effects, can inhibit the transformation of lung adenocarcinoma, and ultimately inhibit the proliferation of lung adenocarcinoma cells.

### 2.2 Leo promotes cancer cell apoptosis

Apoptosis, delineated as a genetically-regulated mechanism of cellular demise, functions as a vital biological process within the human body, mediating the systematic removal of damaged and aberrant cells (Darcy, 2019; Kornepati et al., 2022; You et al., 2023c). This intrinsic cellular mechanism operates as a natural impediment, restraining the endurance and propagation of malignant cellular entities (Cheung and Vousden, 2022; Matthews et al., 2022; You et al., 2023d). Notwithstanding, cancer cells ingeniously circumvent the apoptosis pathway, by inducing genetic mutations or eliciting epigenetic alterations therein, thereby accentuating the significance of harnessing the apoptosis pathway for the eradication of cancer cells in therapeutic oncology (Chen et al., 2013b; Ozyerli-Goknar and Bagci-Onder, 2021; Karami Fath et al., 2022; Jin and Jeong, 2023).

Apoptotic signal transduction fundamentally encompasses both exogenous and endogenous mechanisms. Intracellular triggers, such as DNA damage, deficiency of growth factors, and oxidative stress predominantly activate the endogenous apoptosis pathway (Rezatabar et al., 2019; Souliotis et al., 2019; Wenqi et al., 2023). Conversely, exogenous apoptosis is principally prompted by the interaction between Fas ligand and Fas receptor or Tumor Necrosis Factor (TNF) ligand and its receptor. Experimental findings suggest that the compound “Leo” has the potential to instigate both these apoptosis pathways in cancer cells. Leo’s impact on the exogenous apoptotic mechanism is a significant aspect to consider. Exogenous apoptosis is mainly initiated by the interaction between death ligands and their corresponding death receptors, such as Fas ligand and Fas receptor or Tumor Necrosis Factor (TNF) ligand and its receptor. Studies have shown that Leo can sensitize cancer cells to the exogenous apoptotic pathway by increasing the expression of death receptors on the cell surface. Leo has been found to upregulate the expression of Death Receptor 4 (DR4) and Death Receptor 5 (DR5), both of which are involved in the initiation of the extrinsic apoptotic pathway. This enhancement in death receptor expression can render cancer cells more susceptible to death ligand-induced apoptosis, further contributing to Leo’s antitumor effects (Huang et al., 2021; Wu et al., 2021; You et al., 2023e; Schramm et al., 2023). Thus, Leo’s influence on both endogenous and exogenous apoptotic pathways underscores its potential as a multifaceted agent for promoting apoptosis in cancer cells. (Zhao et al., 2021; Zhang et al., 2022; Zhou et al., 2022).

### 2.3 Leo promotes autophagy in cancer cells

Autophagy represents a universal metabolic degradation mechanism that encompasses the inclusion of intracellular cytoplasmic proteins or organelles within vesicular structures. These subsequently amalgamate with lysosomes to form autolysosomes, which serve the function of degrading the sequestered material (Brennand et al., 2011; Joshi et al., 2020; Vargas et al., 2023). The byproducts of this degradation process are repurposed to facilitate metabolic adaptation and uphold energy equilibrium, hence ensuring cellular metabolic processes and rejuvenation. In certain circumstances, autophagy within the cells can precipitate cell death. Atg12, an instrumental gene implicated in autophagy, governs the generation of autophagy precursors and the establishment of autophagosomes, thereby modulating cellular autophagy. A multitude of studies have demonstrated an inhibition of Atg12 in an array of cancer cells.

Post the administration of Leo to hepatocellular carcinoma cell lines, evidence of autophagosome formation and heightened levels of LC3-II were documented within the cells, corroborating the induction of autophagy in the Leo-treated hepatocellular carcinoma cells. In addition, various investigations have indicated the dual capability of Leo to trigger both autophagy and apoptosis concurrently in diverse cancer cells. It is noteworthy that the Leo concentration bears an impact on the incidence rates of both apoptosis and autophagy. When the concentration of Leo is less than 150 μmol·L-1, the rate of autophagy induction is superior to that of apoptosis. However, at Leo concentrations surpassing 150 μmol·L-1, the apoptosis incidence rate surpasses that of autophagy (Zhao et al., 2021; Meng et al., 2023; Zhou et al., 2023).

### 2.4 Leo inhibits immune evasion in cancer cells

Cancer immune surveillance is an important process where the immune system monitors, recognizes, and eliminates nascent cancer cells. Initially, both innate and adaptive immune responses can regulate cancer growth (Chavez-Dominguez et al., 2021; Peña-Romero and Orenes-Piñero, 2022). In the elimination phase, the progression of cancer triggers an acute inflammatory response, initiating the recognition of tumor cells, secretion of pro-inflammatory cytokines, and killing of tumor cells by innate immune cells. Subsequently, dendritic cells migrate to nearby lymph nodes, presenting tumor antigens and activating tumor-specific CD4^+^ and CD8^+^ T cells, which then migrate to the tumor site to kill the cancer cells. The final outcomes are either the complete eradication of tumor cells or the development of resistant clonal variants.

If the latter occurs, the clonal variants can develop resistance by lowering their immunogenicity or secreting and recruiting immunosuppressive factors, thus entering the immune escape stage. Therefore, inhibiting immune evasion of tumor cells can be an effective means of treating lung cancer. Multiple studies have shown that Leonurine can inhibit bladder cancer’s immune evasion and prolong patients' survival. Many clinical studies show that Leonurine inhibits lung cancer cell immune evasion and exerts anti-lung cancer effects by inhibiting the ILT4-PI3K/AKT-B7-H3 pathway in human lung cancer cells and down-regulating ILT4, PI3K, AKT, B7-H3 mRNA, and protein expression (Stroe and Oancea, 2020; Laurindo et al., 2023). Cytotoxic T lymphocyte-associated antigen 4 (CTLA-4) can inhibit T lymphocyte activation and subsequently cause immune evasion in the tumor microenvironment. Wang et al.’s study (Wang et al., 2022) suggests that Leonurine can inhibit CTLA-4 expression, reversing CTLA-4 mediated immune evasion.

### 2.5 Leo can inhibit tumor migration and invasion

Cell invasion and metastasis refer to the process where cells from the primary site grow and proliferate in another location through blood and lymph (Fares et al., 2020; Paço et al., 2020; Bui et al., 2021). Cancer in its early stages is prone to invasion and metastasis, which are significant biological behaviors of cancer cells. Domestic and international scholars have confirmed that Leo has a significant anti-metastatic effect on various cancer cells. The key issue for the invasion and metastasis of cancer cells is the degradation of extracellular matrix components by matrix metalloproteinases (MMPs).

Multiple studies have shown that Leo can significantly inhibit the invasion and metastasis capabilities of hepatocellular carcinoma Bel-7402 cells *in vitro*. The molecular mechanism may be through reducing the expression levels of matrix metalloproteinase-2 (MMP-2) and matrix metalloproteinase-9 (MMP-9) in hepatocellular carcinoma Bel-7402 cells, reducing the degradation of the extracellular matrix and basement membrane, and decreasing the cells' invasion and metastasis ability. A considerable amount of research shows that in in vitro experiments, Leo can inhibit transcription factor AP1 in Hep G2 hepatocellular carcinoma cells through the Mitogen-Activated Protein Kinase (MAPK) signaling pathway, reduce the expression of matrix metalloproteinase-3 (MMP-3), thereby significantly reducing the number of cells passing through the polycarbonate membrane and effectively inhibiting cell invasion and metastasis. This further proves that Leo inhibits the activity of matrix metalloproteinases MMPs, reducing the degradation of extracellular matrix components and inhibiting the invasion and metastasis of liver cancer cells (Gao et al., 2022; Kumar et al., 2019; Zhang et al., 2023).

## 3 Tumor cellular signaling pathways and mechanisms affected by leo

### 3.1 Leo-mediated NF-κB signaling pathway and fucosyltransferase IV (FUT4) in lung cancer treatment

Lung carcinoma represents the malignancy category with the highest mortality incidence on a global scale. The hypoxic microenvironment intrinsic to lung tumors frequently catalyzes the epithelial-mesenchymal transition (EMT) and promotes cancer cell stemness, thus imparting substantial impediments to efficacious lung carcinoma treatment (Tsoukalas et al., 2017; Wa et al., 2022). A critical determinant influencing treatment prognoses is the diminution of sensitivity towards cisplatin-based chemotherapeutics. Empirical studies have demonstrated that the strategic pairing of cisplatin with Leo can effectively attenuate hypoxia-activated NF-κB signaling pathways and their consequent mediation of EMT and stemness, thereby enhancing both *in vivo* and *in vitro* responsiveness to cisplatin chemotherapies. Within the purview of lung carcinoma, both FUT4 and its synthesized cancer-specific glycoantigen, Lewis Y (LeY), are aberrantly elevated, with FUT4 demonstrating notable overexpression within lung carcinogenic tissues.

The effects of Leo are discerned to be dose- and time-dependent, commanding a marked downregulation of FUT4 expression, which sequentially results in a decrease in LeY biosynthesis, impedes EGFR activation via a reduction in the synthesis of LeY-laden EGFR, and hinders the MAPK and NF-κB signal transduction pathways. These events collectively inhibit neoplastic cellular migration, invasion, and EMT. Consequently, Leo may function as a crucial pharmaceutical agent inhibiting EMT by targeting FUT4 in lung carcinoma, possessing the potential to impede tumor proliferation by suppressing EGFR and its downstream signaling pathways. Investigation of LeoR’s inhibitory effects on lung carcinoma migration, invasion, and resistance to anoikis have unveiled its aptitude to significantly enhance the expression of epithelial marker proteins during the TGF-β1 induction process. This implies that Leo’s capacity to inhibit the EMT process can mitigate lung tumor migration and invasion, thus potentially positioning Leo as a novel antimetastatic pharmaceutical candidate for lung carcinoma treatment, with the expectation of its integration into mainstream comprehensive treatment of lung carcinoma (Wang et al., 2021; Zhu et al., 2021; Wu et al., 2023).

### 3.2 Leo inhibits PI3K/AKT-related signaling pathways

The phosphatidylinositol 3-kinase (PI3K)/protein kinase B (AKT) and mammalian target of rapamycin (mTOR) signaling axes seamlessly collaborate in the orchestration of a myriad of cellular functions including growth, proliferation, metabolic processes, and gene transcription (Marquard and Jücker, 2020; Tan, 2020). PI3K, a pivotal component of the mTOR/AKT pathway, is strategically situated downstream of the receptor tyrosine kinase (RTK). AKT, prominently positioned as one of the most hyperactivated proteins implicated in a diverse array of carcinomas, experiences excessive activation due to an assortment of factors. These factors encompass, but are not limited to, mutation and deletion events in the tumor suppressor gene PTEN. Leo has the ability to interact with various proteins in the mTOR/PI3K signaling axis, as well as proteins affiliated with this pathway.

Leo interacts with the ATP binding site of PI3K, thereby inhibiting the assembly of the mTORC2 complex. This interaction further suppresses the phosphorylation of AKT at Ser473, ultimately leading to the inhibition of the PI3K/AKT signaling pathway. Additionally, Leo has been observed to induce the activation of adenosine monophosphate-activated protein kinase (AMPK) in conjunction with Calcium/Calmodulin-dependent protein kinase β (CaMKKβ) and another unidentified upstream kinase of AMP-dependent protein kinase. This action results in the augmented functionality of Tuberous Sclerosis Complex 2 (TSC2) via promotion of its phosphorylation, thereby further curtailing mTOR activity to exercise its anti-neoplastic effect (Sun et al., 2020; Hou et al., 2022; Xiang et al., 2023).

### 3.3 Leo controls cancer invasion and metastasis by inhibiting EMT

The instigation of epithelial-mesenchymal transition (EMT) transcription factors, or the elicitation of EMT itself, can potentiate stem-like properties in neoplastic cells. This conversion into cancer stem cells has been identified as a principal mechanism propelling cancer recurrence (Toh et al., 2017; Hong et al., 2018). Specifically pertaining to colorectal neoplasia, the colorectal stem cells have been identified as the progenitor entities underpinning colorectal cancer’s invasion and metastasis.

Leo presents a substantial capacity to depress the expression of stem genes and EMT markers within colorectal cancer cells. This repression, driven through a Snail-dependent pathway, engenders the inhibition of colorectal cancer cells’ migratory capacity, while diminishing both the quantity and magnitude of neoplastic nodes. This underscores the potential clinical utility of Leo in the therapeutics of colorectal cancer.

In the context of nasopharyngeal carcinoma, distant metastasis is frequently implicated as a primary cause of therapeutic failure. However, Leo has demonstrated potential to impede EMT through the regulation of Matrix Metalloproteinases-2 and -9 (MMP-2 and MMP-9), resulting in the inhibition of nasopharyngeal carcinoma cell migration and invasion. Cumulatively, these studies affirm the potential of Leo as a therapeutic agent in the management of tumor invasion and metastasis (Hu et al., 2019; Zheng et al., 2021).

### 3.4 Leo activates tumor necrosis factor-related apoptosis-inducing ligand (TRAIL) signaling pathway

The Tumor Necrosis Factor Related Apoptosis-Inducing Ligand (TRAIL) possesses the capability to initiate apoptosis in neoplastic cells, while generally demonstrating negligible toxicity to the majority of normal cellular entities. Anomalies in the TRAIL signalling cascade are implicated in contributing to chemotherapeutic resistance and anti-apoptotic mechanisms prevalent within an array of neoplastic cell types. The compound Leo exhibits a capacity to directly engage with Adenine Nucleotide Translocator 2 (ANT-2), an inhibitor of TRAIL, thereby facilitating an upregulation of TRAIL’s activity through suppression of ANT-2 expression. This interaction prompts the induction of apoptosis within colorectal carcinoma cells (Jan and Chaudhry, 2019; Carneiro and El-Deiry, 2020; Mirzapour et al., 2023).

Concurrently, Leo manifests a noteworthy propensity to augment the protein expression degrees of Death Receptor 4 and Death Receptor 5. These receptors demonstrate specificity towards TRAIL binding, engendering tumour cell apoptosis via the transmission of apoptotic pathways. Alongside this, Leo prompts a modification in the alternative splicing of the TRAIL death-inducing signalling complex within pulmonary carcinoma cells. This modulation, in conjunction with Leo’s capacity to bind to Heat Shock Protein 70, ultimately results in an enhancement of TRAIL pathway activity. Notably, in TRAIL-resistant neoplastic cells such as A549, Leo retains its ability to curtail the expression level of NF-κB, leading to an inhibition of various anti-apoptotic proteins.

Leo’s anti-neoplastic influence is exerted through the “Leo-microRNA-TRAIL” interaction, a mechanism indicative of Leo’s significant potential in the design of clinical oncological therapeutics (Zong and Zhao, 2021; Han et al., 2022).

### 3.5 Leo inhibits signal transducer and activator of transcription 3 (STAT3) related signaling pathway

The Janus Kinase/Signal Transducer and Activator of Transcription (JAK/STAT) signaling cascade is a critical conduit in the orchestration of developmental processes, mediating a plethora of activities including cellular migration, apoptosis, proliferation, and the initiation of inflammatory responses. The Signal Transducer and Activator of Transcription (STAT) forms a crucial part of this molecular architecture, with STAT3 representing a specific element within the STAT family. When phosphorylated, STAT3 proteins engage in interaction via SH-2 structural domains, leading to their dimerization. Following this, the dimerized structures traverse to the nucleus, aided by importin and Ran, in order to exert influence over gene transcription (Xin et al., 2020; Hu et al., 2021).

In the milieu of neoplastic cellular landscapes, STAT3 is characterized by an anomalously persistent activation. Leo is noted for its capacity to engage in an interaction with two key kinases: JAK and the non-receptor tyrosine kinase, SRC. This molecular dialogue results in the attenuation of their phosphorylation activity, a process that subsequently inhibits the phosphorylation of the STAT3 protein. Consequently, this serves to obstruct both the dimerization and nuclear translocation of STAT3, thus instigating an anti-neoplastic influence. In the paradigm of HER2-overexpressing breast cancer cell lines, namely SKBR3 and MDAMB-453, application of Leo has been empirically demonstrated to diminish the expression of STAT, JAK2, and Vascular Endothelial Growth Factor (VEGF). This empirical evidence advocates for Leo’s inhibitory action on the STAT/VEGF signaling cascade, ultimately leading to the suppression of tumor cell proliferation, invasiveness, and metastasis. Moreover, findings derived from clinical investigations disclose Leo’s capability to obstruct the secretion of inflammatory cytokines via interference with the STAT3/NF-κB signaling axis, in addition to reducing the expression of oncogenic markers such as p53 and CEA. This unveils the prospective dual nature of Leo, possessing both anti-inflammatory and anti-neoplastic faculties, with a potential contribution towards halting the evolution from colitis to colon cancer. In the arena of drug-resistant neoplasms, scholarly research suggests that Leo can depress the expression of resistance-associated proteins present in drug-resistant lung adenocarcinoma cells and inhibit cellular proliferation by stifling STAT3 activation, thus combating drug resistance. This provides a testament to Leo’s capacity to exert an anti-neoplastic influence on tumor cells, especially those displaying drug resistance, via the modulation of STAT3-related signaling pathways (Kim et al., 2019; Shi et al., 2022). In support of Leo’s efficacy in inhibiting the STAT3 signaling pathway, several studies have corroborated these findings. For instance, (Dattilo et al., 2020), demonstrated that Leo treatment led to a substantial reduction in phosphorylated STAT3 levels in hepatocellular carcinoma cells, consequently impeding their proliferation and invasiveness. Similarly, (Liu et al., 2021), reported that Leo treatment effectively suppressed the activation of STAT3 and its downstream target genes in pancreatic cancer cells, leading to a notable inhibition of tumor growth. Furthermore, (Zhang et al., 2020), documented the downregulation of phosphorylated STAT3 in glioblastoma cells treated with Leo, which was accompanied by a decrease in cell migration and invasion. These studies collectively underscore Leo’s potential as a promising candidate for targeting the STAT3 pathway in diverse cancer types, emphasizing its role in inhibiting tumor progression.

### 3.6 Leo inhibits mitogen-activated protein kinase (MAPK) related pathway

The MAPK cascade is an intricate cellular process which comprises three successive activation kinases: Raf, Mek, and Extracellular Signal-regulated Kinase (ERK) (Chin et al., 2019; Guo et al., 2020; Pan et al., 2022). The cascade is set into motion when Ras, a category of G-protein, instigates a chain reaction, sequentially activating Raf, Mek, and ERK. The latter, ERK, proceeds to exert influences on both upstream and downstream targets within the cell.

This signaling pathway is instrumental in the modulation of a myriad of crucial cellular processes, including, but not limited to, cell proliferation, differentiation, and programmed cell death. In a significant majority of cancer variants, MAPK signaling is persistently activated, an aberration attributable to an array of factors. These include overexpression of cellular receptors, mutations in the Ras and Raf genes, and activation mutations of Receptor Tyrosine Kinase (RTK).

Several studies have reported the regulatory role of Leo on the MAPK/ERK pathway across a variety of cancers. For instance, in non-small cell lung cancer cells, Leo has been demonstrated to inhibit the activation of AKT, ERK, and NF-κB, thereby amplifying TRAIL-induced apoptosis. Furthermore, it has been observed to restrain the phosphorylation levels of ERK1/2, AKT, and mTOR, triggering a standstill at the G2/M phase, instigating apoptosis, and thus repressing the proliferation, invasion, and migration of human melanoma cell lines A375 and C8161.

In the context of human melanoma cells, Leo induces anoikis, a form of programmed cell death, by downregulating integrin levels, inhibiting the phosphorylation of FAK and ERK1/2, and thus suppressing tumor cell migration. In gastric cancer cells, it diminishes cell survival and incites apoptosis by impeding the AKT/mTOR pathway while concurrently escalating the phosphorylation levels of ERK1/2 and P90RSK in a dose-dependent manner (Patrignani et al., 2020; Salama et al., 2022).

## 4 Prospects

Leo is identified as a principal active alkaloid in motherwort, possessing an extensive spectrum of pharmacological activities. This includes various effects, such as anti-inflammatory and anti-oxidative properties. Leo has the capability to inhibit the proliferation, migration, and invasion of tumor cells, along with the induction of apoptosis and autophagy, by influencing diverse signaling pathways. This demonstrates significant anti-tumor effects, which have been observed in both *in vitro* and *in vivo* studies. The commendable anti-tumor properties of Leo, coupled with its relatively minimal side effects and economical cost, position it as a promising candidate for further exploration and development in the field of anti-tumor pharmaceuticals. While the role of active ingredients from traditional Chinese medicine in anti-tumor treatment is gaining increasing attention, it is essential to examine the potential advantages of these ingredients in comparison to established treatment modalities such as traditional chemoradiotherapy or targeted therapy. Leo offers several potential advantages over conventional treatments. Unlike chemoradiotherapy, which often exhibits substantial toxicity to healthy tissues and cells, Leo has shown relatively minimal side effects in preclinical studies, suggesting a more favorable safety profile. Moreover, Leo’s multi-faceted mechanisms of action, including its modulation of various signaling pathways, could potentially make it effective against tumors that exhibit resistance to single-targeted therapies. This broad-spectrum approach might be particularly beneficial in treating heterogeneous cancers with complex genetic landscapes. Additionally, Leo’s natural origin could provide an alternative treatment avenue for patients who may not tolerate or respond well to existing therapies. However, it’s important to note that while these advantages hold promise, further comprehensive clinical investigations are necessary to validate the true potential of Leo as a valuable addition to the existing arsenal of anti-tumor treatments. While this review has extensively covered the wide range of anti-tumor effects of Leo and its impact on various signaling pathways, there is a need for further exploration to elucidate the specific molecular targets through which Leo exerts its effects. While the mechanisms described shed light on Leo’s influence on apoptosis, autophagy, proliferation, and migration, identifying the precise molecular interactions that drive these processes will enhance our understanding of its pharmacological activities. Future research endeavors should focus on uncovering the exact targets of Leo within the signaling pathways mentioned. By providing detailed insights into the specific molecular interactions and pathways influenced by Leo, we can not only enrich our comprehension of its anti-tumor potential but also pave the way for targeted drug development and clinical translation. This emphasis on precise molecular targets will allow for a more comprehensive and focused exploration of Leo’s therapeutic applications in the realm of anti-tumor treatments. Current research has proven that Leo can exert anti-tumor effects by regulating various pathways, but reports of Leo being used for clinical tumor treatment are still limited, and no research has been done on using Leo to make anti-tumor drugs. Future research should continue to advance in the following three areas:

1. On the basis of clarifying the pathways, explore the safety and effectiveness of Leo for clinical use.
2. Clarify the pharmacological mechanism of Leo by validating its target of action.
3. Explore issues related to Leo’s solubility and bioavailability to validate its drugability.

## 5 Conclusion

The pharmacological effects and mechanisms of Leo are receiving more and more attention. This article introduces the anti-tumor mechanisms of Leo and related signaling pathways, pointing out that Leo exerts its anti-tumor effect by inducing apoptosis and autophagy in tumor cells, inhibiting tumor cell proliferation, invasion, and migration, and introduces the signaling pathways and mechanisms of the tumor cells affected by Leo. Ultimately, it was found that Leo can exert its inhibitory effect on tumor cell proliferation, migration, and invasion, induce apoptosis and autophagy, by affecting various signaling pathways, making it a molecule with high research value and potential for anti-tumor properties. Future research on Leo should focus on exploring the clinical safety and effectiveness of Leo, validating the targets of Leo’s action and elucidating the pharmacological mechanisms of Leo, while also validating Leo’s drugability.
